# A cysteine proteinase inhibitor ALLN alleviates bleomycin-induced skin and lung fibrosis

**DOI:** 10.1186/s13075-023-03130-7

**Published:** 2023-08-25

**Authors:** Hiroshi Kasamatsu, Takenao Chino, Takumi Hasegawa, Natsuko Utsunomiya, Akira Utsunomiya, Masami Yamada, Noritaka Oyama, Minoru Hasegawa

**Affiliations:** 1 Department of Dermatology, Division of Medicine, Faculty of Medical Sciences, University of Fukui, 23-3 Matsuoka-Shimoaizuki, Eiheiji-Cho, Yoshida-Gun, Fukui, 910-1193 Japan; 2 Department of Cell Biology and Biochemistry, Division of Medicine, Faculty of Medical Sciences, University of Fukui, 23-3 Matsuoka-Shimoaizuki, Eiheiji-Cho, Yoshida-Gun, Fukui, 910-1193 Japan

**Keywords:** Systemic sclerosis, Skin, Lung, Calpain, Cysteine proteinase inhibitor, TGF-β, Fibrosis, Endothelial-mesenchymal transition

## Abstract

**Background:**

Systemic sclerosis (SSc) is a connective tissue disease that is characterized by fibrosis in the skin and internal organs, such as the lungs. Activated differentiation of progenitor cells, which are mainly resident fibroblasts, into myofibroblasts is considered a key mechanism underlying the overproduction of extracellular matrix and the resultant tissue fibrosis in SSc. Calpains are members of the Ca^2+^-dependent cysteine protease family, whose enzymatic activities participate in signal transduction and tissue remodeling, potentially contributing to fibrosis in various organs. However, the roles of calpain in the pathogenesis of SSc remain unknown. This study aimed to examine the anti-fibrotic properties of N-acetyl-Leu-Leu-norleucinal (ALLN), one of the cysteine proteinase inhibitors that primarily inhibit calpain, in vitro and in vivo, to optimally translate into the therapeutic utility in human SSc.

**Methods:**

Normal human dermal and lung fibroblasts pretreated with ALLN were stimulated with recombinant transforming growth factor beta 1 (TGF-β1), followed by assessment of TGF-β1/Smad signaling and fibrogenic molecules.

**Results:**

ALLN treatment significantly inhibited TGF-β1-induced phosphorylation and nuclear transport of Smad2/3 in skin and lung fibroblasts. TGF-β1-dependent increases in α-smooth muscle actin (αSMA), collagen type I, fibronectin 1, and some mesenchymal transcription markers were attenuated by ALLN. Moreover, our findings suggest that ALLN inhibits TGF-β1-induced mesenchymal transition in human lung epithelial cells. Consistent with these in vitro findings, administering ALLN (3 mg/kg/day) three times a week intraperitoneally remarkably suppressed the development of skin and lung fibrosis in a SSc mouse model induced by daily subcutaneous bleomycin injection. The number of skin- and lung-infiltrating CD3^+^ T cells decreased in ALLN-treated mice compared with that in control-treated mice. Phosphorylation of Smad3 and/or an increase in αSMA-positive myofibroblasts was significantly inhibited by ALLN treatment on the skin and lungs. However, no adverse effects were observed.

**Conclusions:**

Our results prove that calpains can be a novel therapeutic target for skin and lung fibrosis in SSc, considering its inhibitor ALLN.

## Introduction

Systemic sclerosis (SSc) is a connective tissue disorder that presents as fibrosis and vascular injury with an autoimmune basis. Although the pathogenesis remains unclear, inflammation, which is primarily of macrophages and T cells, precedes excessive extracellular matrix production, such as collagen from myofibroblasts, and tissue deposition [1, 2]. In this process, profibrogenic molecules, such as transforming growth factor-beta 1 (TGF-β1), produced by immune and mesenchymal cells induce the differentiation of resident fibroblasts and other progenitor cells into myofibroblasts. Recently, various agents that inhibit these mechanisms have been validated as therapeutic agents [3–5]. However, the treatments for SSc are still limited and unestablished.

Calpains are members of the Ca^2+^-dependent cysteine proteases that catalyze the controlled proteolysis of specific substrates and play various critical roles, including cell growth, differentiation, and apoptosis [6]. Many previous reports indicate that calpains are potential therapeutic targets for various disorders, including cancers, fibrosis, ophthalmic diseases, neurodegenerative disorders, cardiovascular disorders, and eosinophilic esophagitis [7–9]. Although clinical trials have been conducted using several calpain inhibitors, none have been translated into practical use.

Humans possess 15 calpain genes, which are from CAPN1 to CAPN16, excluding CAPN4. Specifically, the two conventional calpains, calpain 1 (μ-calpain) and calpain 2 (m-calpain), are the most ubiquitously expressed and well-studied. They alter the structure and functionality of their substrates through limited proteolysis. However, discrepancies exist between in vivo and in vitro findings regarding the functionality of calpain 1 and 2. Because calpain 1 and calpain 2 can be activated by micro- and milli-molar concentrations of Ca^2+^, respectively, calpain 1 is more widely used in vivo [6]. However, CAPN1-deficient mice are apparently normal [10]. In contrast, the loss of CAPN2 leads to embryonic lethality [11, 12], indicating that calpain 2 plays a more crucial role than calpain 1 in vivo.

Previous studies have shown that calpain contributes to the development of fibrosis and tissue remodeling. For example, calpain 1 and 2 expression increased in the thickened pulmonary interalveolar septa of mouse lung tissues treated intraperitoneally with bleomycin injection [13]. In this model, calpeptin, which is a cysteine proteinase inhibitor that hinders calpain 1, calpain 2, and papain, prevented the development of fibrosis by suppressing proinflammatory and profibrotic cytokines production such as interleukin-6 (IL-6), TGF-β1, angiopoietin-1 (Ang-1), and collagen I synthesis. Furthermore, calpeptin inhibited proliferation, migration, collagen synthesis, and TGF-β1 production in human lung fibroblasts in vitro. Another study reported that intraperitoneal injections of bleomycin protected calpain-1/2 conditional knockout mice against lung fibrosis [14]. In that study, calpain activation and TGF-β1 interacted to increase collagen I synthesis in human lung fibroblasts. However, the precise mechanism of calpain-dependent fibrosis and the potential use of its inhibitors in SSc treatment remain unclear.

Therefore, we aimed to examine the use of N-acetyl-Leu-Leu-norleucinal (ALLN), which is a cysteine proteinase inhibitor that impedes mainly calpain 1 and calpain 2, and cysteine cathepsins, both in vitro and in vivo. ALLN treatment inhibited the TGF-β1-induced differentiation of skin and lung fibroblasts into myofibroblasts and subsequent extracellular matrix (ECM) production in vitro. In addition, peritoneally injecting ALLN attenuated the development of skin and lung fibrosis, which was induced by the subcutaneous injection of bleomycin in a mouse model.

## Materials and methods

### Fibroblasts

Normal human fetal foreskin fibroblasts were purchased (Kurabo Industries, Osaka, Japan); normal lung fibroblasts from the fetal lung were grown in Dulbecco’s modified Eagle’s medium (DMEM) (Nacalai Tesque, Kyoto, Japan) containing 10% fetal bovine serum (FBS) (Biosera, Kansas City, MO, USA) and 1% antibiotics (100 U/mL each of penicillin and streptomycin) (Nacalai Tesque) in a humidified incubator with 5% CO_2_ at 37 °C. When reached approximately 70% confluence, the cells were starved in DMEM containing 0.1% FBS for 24 h and subsequently pretreated with dimethyl sulfoxide (DMSO) as control or various concentrations of the calpain inhibitor, ALLN (Sigma-Aldrich, St. Louis, MO, USA) (diluted in DMSO). One hour later, the cells were stimulated with 10 ng/mL recombinant human TGF-β1 (PeproTech, Rocky Hill, NJ, USA).

### Epithelial cells

A human non-small cell lung carcinoma cell line A549 cells (American Type Culture Collection) were grown in minimum essential medium (MEM, Sigma-Aldrich) containing 10% FBS and 1% antibiotics in a humidified incubator with 5% CO_2_ at 37°C. The medium was replaced with 5% FBS-containing MEM when the cells reached approximately 70% confluence. Next, cells were pretreated with DMSO as control or various concentrations of ALLN (Sigma-Aldrich) diluted in DMSO for 30 min and subsequently stimulated with 10 ng/mL recombinant human TGF-β1 (PeproTech).

### Bleomycin-induced fibrosis mouse model (animal protocol)

This study used female C57BL/6J mice aged between 8 and 10 weeks (CLEA Japan, Tokyo, Japan). Mice were subcutaneously injected with 150 μL bleomycin (1 mg/mL) or 150 μL saline in the control group into the shaved back daily for 4 weeks, as previously described [15]. Concurrently, ALLN (3 mg/kg/day) or phosphate-buffered saline (PBS) was administered intraperitoneally three times weekly. The ALLN dose was determined in a pilot study. Finally, the mice were euthanized on day 28, and samples were collected to assess skin and lung fibrosis.

### Hematoxylin and eosin and Masson’s trichrome staining

Briefly, the mouse skin and lungs were fixed in 10% formalin and embedded in paraffin, followed by histological assessment using a light microscope. All skin sections were obtained from the back with scalpels and lungs from the right middle lobe. The sections were stained with hematoxylin and eosin (H&E) and Masson’s trichrome. Dermal thickness was defined as the skin thickness from the epidermal-dermal junction to the junction between the dermis and the subcutaneous fat [16]. Two investigators (HK and TC) conducted a blinded examination of the sections. Collagen accumulation was assessed through pixelization of Masson’s trichrome staining area using Photoshop software (ver. 16).

### Collagen assay

Type I collagen in the culture supernatant was measured using an ELISA kit specific for human type I collagen (ACEL, Inc. Kanagawa, JAPAN) according to the protocol. Total collagen accumulation in the skin tissue was assessed using Sirius Red/Fast Green Collagen Staining Kit (Chondrex, Inc., Woodinville, WA, USA) [17]. In brief, paraffin-embedded sections were deparaffinized and were loaded 0.2–0.3 mL Dye Solution and incubated at room temperature for 30 min. After rinsing the stained tissue section, Dye Extraction Buffer was loaded on each sample and gently mixed. The OD values was measured at 540 nm and 605 nm with a spectrophotometer after collecting the eluted Dye Solution.

### RNA preparation and real-time qRT-PCR

Total RNA was isolated from the cultured cells using RNeasy spin columns (Qiagen, Valencia, CA, USA). After reverse transcription into complementary DNA using PrimeScript RT Master Mix system (TaKaRa Bio, Shiga, Japan), real-time quantitative reverse transcription-polymerase chain reaction (qRT-PCR) was performed using a StepOnePlus Real-Time PCR System (Thermo Fisher Scientific, Waltham, MA, USA). Gene transcripts were normalized to glyceraldehyde-3-phosphate dehydrogenase (GAPDH) mRNA expression and quantified as relative expression levels.

### Immunofluorescence

The cells prepared as described above were washed with PBS, fixed with 4% paraformaldehyde phosphate (PFA) for 10 min, and permeabilized with 0.2% Triton X-100 for 3 min. Subsequently, the sections were incubated for 24 h at 4°C with primary antibodies and incubated with fluorescein-conjugated secondary antibodies for 1 h at room temperature. The primary antibodies used were anti-collagen type I alpha 2 (COL1A2) (1:200, Abcam, Cambridge, UK), anti-fibronectin 1 (FN1) (1:200, Abcam), anti-alpha smooth muscle actin (αSMA) (1:200, Abcam), anti-p-Smad3 (1:200, Cell Signaling Technology, USA), anti-snail family transcriptional repressor 2 (SNAIL2) (1:200, Invitrogen, Carlsbad, CA, USA), and anti-zinc-finger-enhancer binding protein 1 (ZEB1) (1:200, Proteintech, Rosemont, IL, USA). Cover glasses were mounted using VECTASHIELD mounting medium with 4,6-diamidino-2-phenylindole (DAPI, Vector Laboratories, Burlingame, CA, USA).

### Immunohistochemistry

Briefly, the mouse skin and lung sections were deparaffinized, and antigen-retrieved by incubating with HistoVT One (Nacalai Tesque) at 90°C for 10 min. Subsequently, the slides were incubated for 10 min with solution to block endogenous peroxidase and alkaline phosphatase (BLOXALL, Vector Laboratories). Next, the slides were immunostained with monoclonal antibodies against mouse CD3 (Adjusted, Nichirei Biosciences) or F4/80 (1:250, Abcam), anti-αSMA antibody (1:200, Abcam), anti-p-Smad3 antibody (1:200, Cell Signaling Technology, USA), anti-calpain 1 antibody (1:200, Abcam), anti-calpain 2 antibody (1:200, Abcam), peroxidase-labeled secondary antibody (Nichirei Biosciences), and a coloration substrate system with 3,3-diaminobenzidine (DAB, Nichirei Biosciences). αSMA- or Calpain-positive area was assessed as follows. One representative section of each mouse was photographed at five random locations in the 200 × field of the microscopic view for analysis. Then, the αSMA- or calpain-positive area was determined through pixelization of the staining area using Photoshop software (ver. 16).

### Western blot analysis

Total protein extraction from human dermal fibroblasts or A549 cells was performed using an extraction kit (101 Bio, Mountain View, CA, USA). Protein concentration was determined using a spectrophotometer and bicinchoninic acid protein assay kit (TaKaRa Bio, Shiga, Japan). Aliquots of protein from each sample were subjected to sodium dodecyl sulfate–polyacrylamide gel electrophoresis on a Mini-PROTEAN TGX Precast gel and transferred to a nitrocellulose membrane (Bio-Rad Laboratories, Hercules, CA, USA). The membrane was incubated with the appropriate primary antibody and fluorescently labeled secondary antibodies. The following antibodies were used: anti-COL1A2 (1:1000, Abcam), anti-FN1 (1:1000, Abcam), anti-αSMA (1:1000, Abcam), anti-p-Smad3 (1:1000, Invitrogen), anti-calpain 1 (1:1000, Abcam), anti-calpain 2 (1:1000, Abcam), anti-E-cadherin (1:1000, Abcam), anti-GAPDH hFAB rhodamine (Bio-Rad Laboratories), and anti-tubulin hFAB rhodamine (Bio-Rad Laboratories). In addition, anti-mouse or anti-rabbit fluorescent-labeled secondary antibodies (all Bio-Rad Laboratories) were used as secondary antibodies. Blots were scanned and quantified using a ChemiDoc imaging system (Bio-Rad Laboratories). The quantified band intensities were normalized to those of GAPDH or tubulin.

### Epithelial-mesenchymal transition (EMT)

A549 human non-small cell lung carcinoma cells (American Type Culture Collection) were maintained in DMEM supplemented with 10% heat-inactivated FBS. Next, EMT was induced in DMEM containing 5% FBS with 10 ng/ml human TGF-β1 recombinant protein (R&D Systems) at each indicated interval, with or without incubation with ALLN, as previously described [18].

### Statistical analysis

Statistical analysis was performed using GraphPad Prism 7 (GraphPad Software Inc., La Jolla, CA, USA). All data are presented as mean ± standard error of the mean, and statistical significance was set at *p* < 0.05, using the Student’s two-tailed *t*-test.

## Results

### ALLN suppressed the TGF-β1-induced expression of fibrogenic molecules in cultured human dermal fibroblasts

**Fig. 1:**
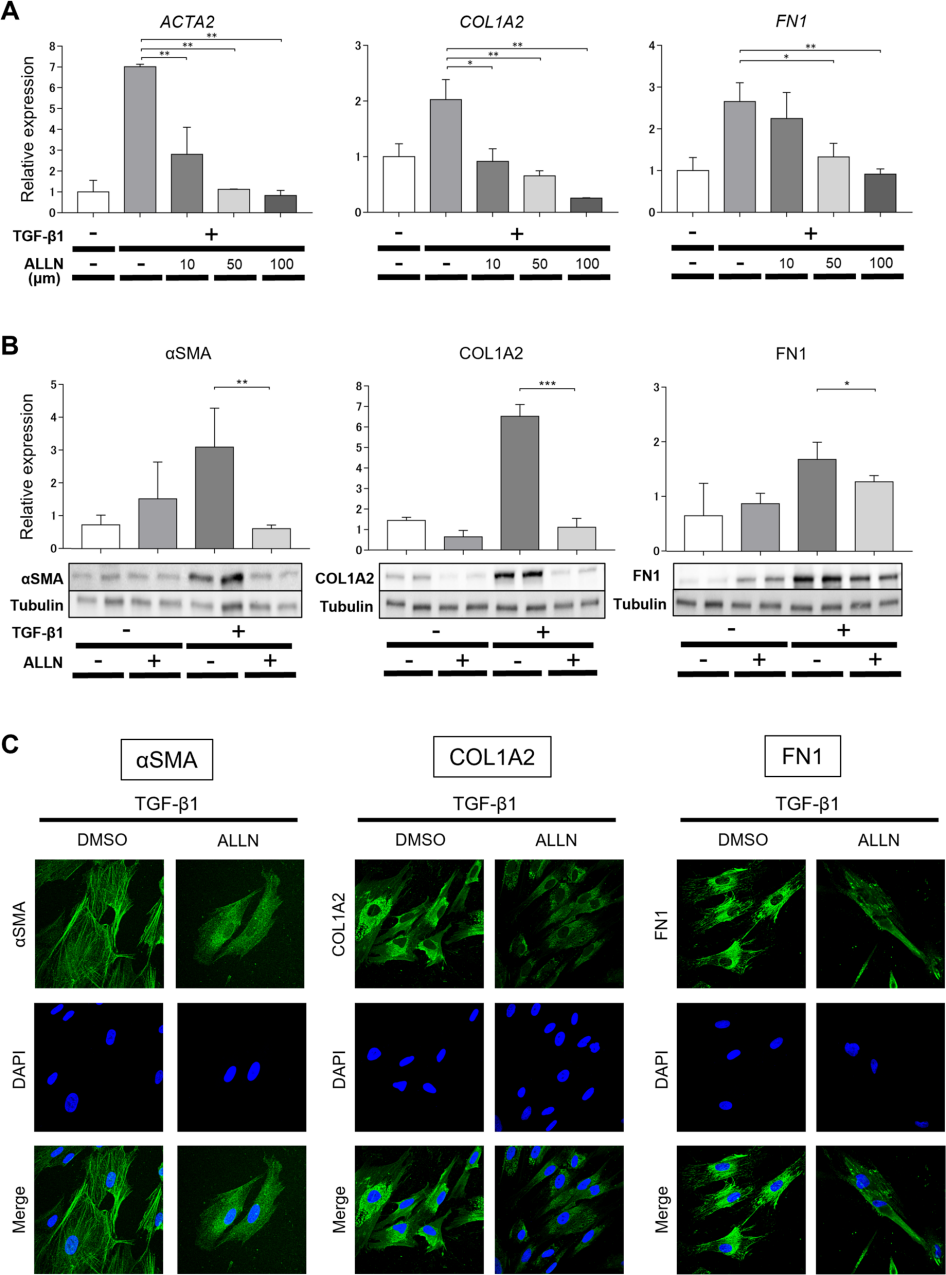
ALLN inhibits the fibrogenic activity of cultured human dermal fibroblasts stimulated with TGF-β1. Normal human dermal fibroblasts were pretreated with DMSO as control or various concentrations of DMSO-diluted ALLN for 1 h, followed by stimulation with 10 ng/ml recombinant human TGF-β1 for an additional 24 h. After harvesting, mRNA and protein expression of the indicated molecules were evaluated by real-time RT-PCR (**A**) and Western blotting (**B**), respectively. Values were normalized to GAPDH and tubulin levels and are shown as relative levels (mean ± SEM). All values represent mean ± SEM; *n* = 5 each group; *, *p* ≤ 0.05; **, *p* ≤ 0.01, ***, *p* ≤ 0.001. **C** Fibroblasts were immunostained for αSMA, COL1A2, or FN1 (*green*). Nuclear counterstaining (*blue*) was performed with DAPI. Representative images of three experiments are shown (400-fold magnification)

**Fig. 2:**
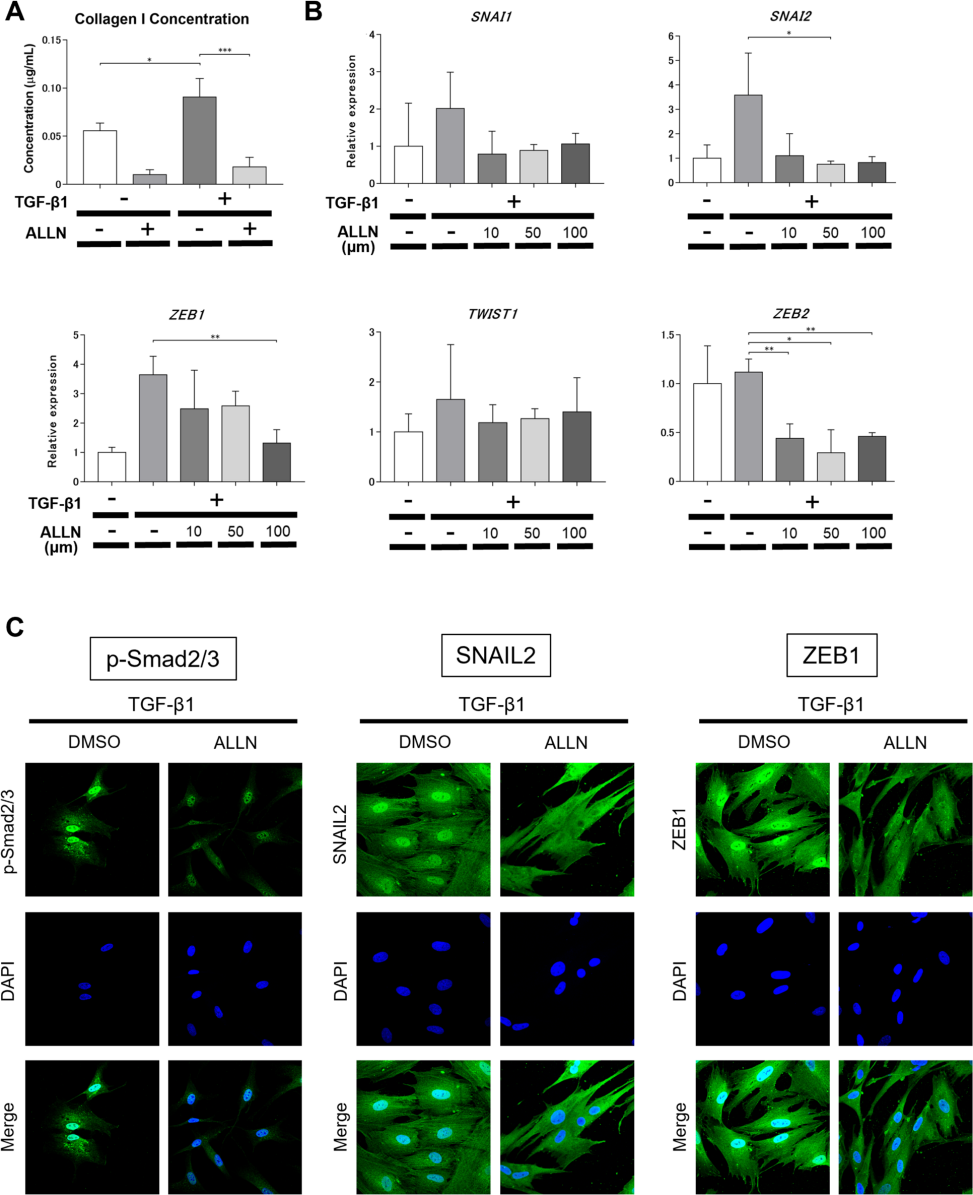
ALLN inhibits the expression of fibrogenic signaling molecules, and transcription factors in cultured human dermal fibroblasts stimulated with TGF-β1. Normal human dermal fibroblasts were pretreated with DMSO as control or various concentrations of DMSO-diluted ALLN for 1 h, followed by stimulation with 10 ng/ml recombinant human TGF-β1 for an additional 24 h. **A** The amount of collagen I in the supernatant was measured by specific ELISA. All values represent the mean ± SEM; *n* = 4 for each group; *, *p* ≤ 0.05; ***, *p* ≤ 0.001. **B** Effects of ALLN on the mRNA expression of transcription factors associated with the mesenchymal transition in human dermal fibroblasts. All values represent the mean ± SEM; *n* = 5 for each group; * *p* ≤ 0.05; ** *p* ≤ 0.01. **C** Fibroblasts were immunostained for phospho-Smad2/3, SNAIL2, or ZEB1 (*green*). Nuclear counterstaining (*blue*) was performed with DAPI. Representative images of three experiments are shown (400-fold magnification)

We investigated the effects of the cysteine proteinase inhibitor, ALLN, on the differentiation of cultured normal human dermal fibroblasts into myofibroblasts and ECM production. The mRNA expression of *ACTA2* (*αSMA*), *COL1A2*, and *FN1* remarkably increased in fibroblasts 48 h after TGF-β1 administration and was significantly inhibited by ALLN dose-dependently (Fig. 1A). Similarly, the TGF-β1-induced protein expression of αSMA, COL1A2, and FN1 was significantly suppressed by ALLN in normal human dermal fibroblasts (Fig. 1B). Immunocytochemistry also showed that ALLN treatment suppressed the TGF-β1-induced cytoplasmic filament formation of αSMA and staining of COL1A2 and FN1 72 h after TGF-β1 stimulation (Fig. 1C). In consistent with these intracellular protein expression, ALLN markedly inhibited collagen I concentration with or without the addition of TGF-β1 in the supernatant of dermal fibroblast culture (Fig. 2A).

### ALLN antagonized the TGF-β1-induced Smad3 phosphorylation and mesenchymal transcription factors in cultured human dermal fibroblasts

TGF-β1 signaling pathway stimulates the expression of mesenchymal transcription factors in fibroblasts. This process involves the phosphorylation and activation of Smad proteins, which translocate into the nucleus and act as transcriptional regulators. Therefore, we evaluated the expression of mesenchymal transcription factors and p-Smad2/3 in TGF-β1-treated normal human dermal fibroblasts to investigate the antifibrotic mechanism of ALLN. The mRNA expression levels of *SNAI2*, *ZEB1*, and *ZEB2* decreased after ALLN treatment in TGF-β1-stimulated fibroblasts at 48 h (Fig. 2B). Immunocytochemical staining showed that ALLN treatment inhibited Smad2/3 phosphorylation at 72 h after TGF-β1 stimulation (Fig. 2C). In addition, ALLN suppressed TGF-β1-induced nuclear translocation of SNAIL2 and ZEB1, which are transcription factors responsible for myofibroblast differentiation (Fig. 2C).

### ALLN inhibited the TGF-β1-induced fibrogenic phenotype in cultured human lung fibroblasts

**Fig. 3:**
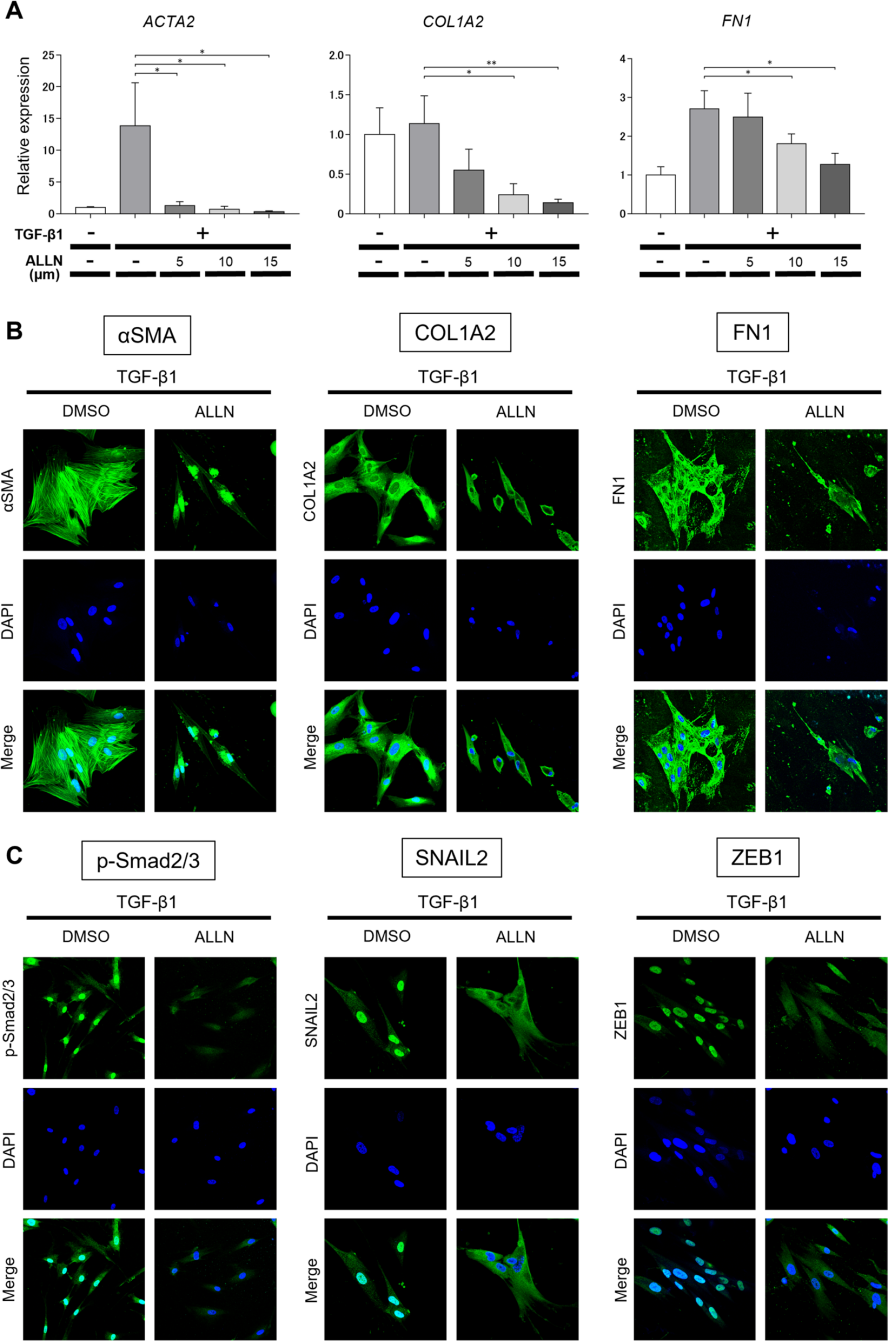
ALLN attenuates the fibrogenic phenotype of cultured human lung fibroblasts stimulated with TGF-β1. Normal human lung fibroblasts were pretreated with DMSO as control or various concentrations of DMSO-diluted ALLN for 1 h, followed by stimulation with 10 ng/ml recombinant human TGF-β1 for an additional 24h. **A** After harvest, mRNA and protein expression of the indicated molecules was evaluated using real-time reverse transcription-polymerase chain reaction (RT-PCR). Values were normalized to GAPDH and are shown as relative levels (mean ± SEM). All values represent mean ± SEM; *n* = 5 each group; *,* p* ≤ 0.05; **, *p* ≤ 0.01, ***,* p* ≤ 0.001. Fibroblasts were also immunostained for αSMA, COL1A2, or FN1 (*green*, **B**) and phospho-Smad2/3, SNAIL2, or ZEB1 (*green*, **C**). Nuclear counterstaining (*blue*) was performed with DAPI. Representative images of three experiments are shown (400-fold magnification)

We also investigated the effect of ALLN on the differentiation of cultured normal human lung fibroblasts into myofibroblasts and on ECM expression. The mRNA expression of *ACTA2* (*αSMA*), *COL1A2*, and *FN1* increased in lung fibroblasts 24 h after TGF-β1 stimulation; however, ALLN generally inhibited this expression (Fig. 3A). Immunocytochemistry showed that ALLN treatment suppressed TGF-β1-induced cytoplasmic filament formation in αSMA (Fig. 3B). In addition, ALLN suppressed COL1A2 and FN1 staining 48 h after TGF-β1 stimulation (Fig. 3B). Moreover, ALLN treatment inhibited the expression and nuclear translocation of phosphorylated Smad2/3, SNAIL2, and ZEB1 at 48 h after TGF-β1 stimulation (Fig. 3C). Therefore, ALLN inhibited calpain-dependent fibrogenic activity and myofibroblast differentiation in normal human lung fibroblasts.

### ALLN suppressed the expression of EMT markers in lung epithelial cells

**Fig. 4:**
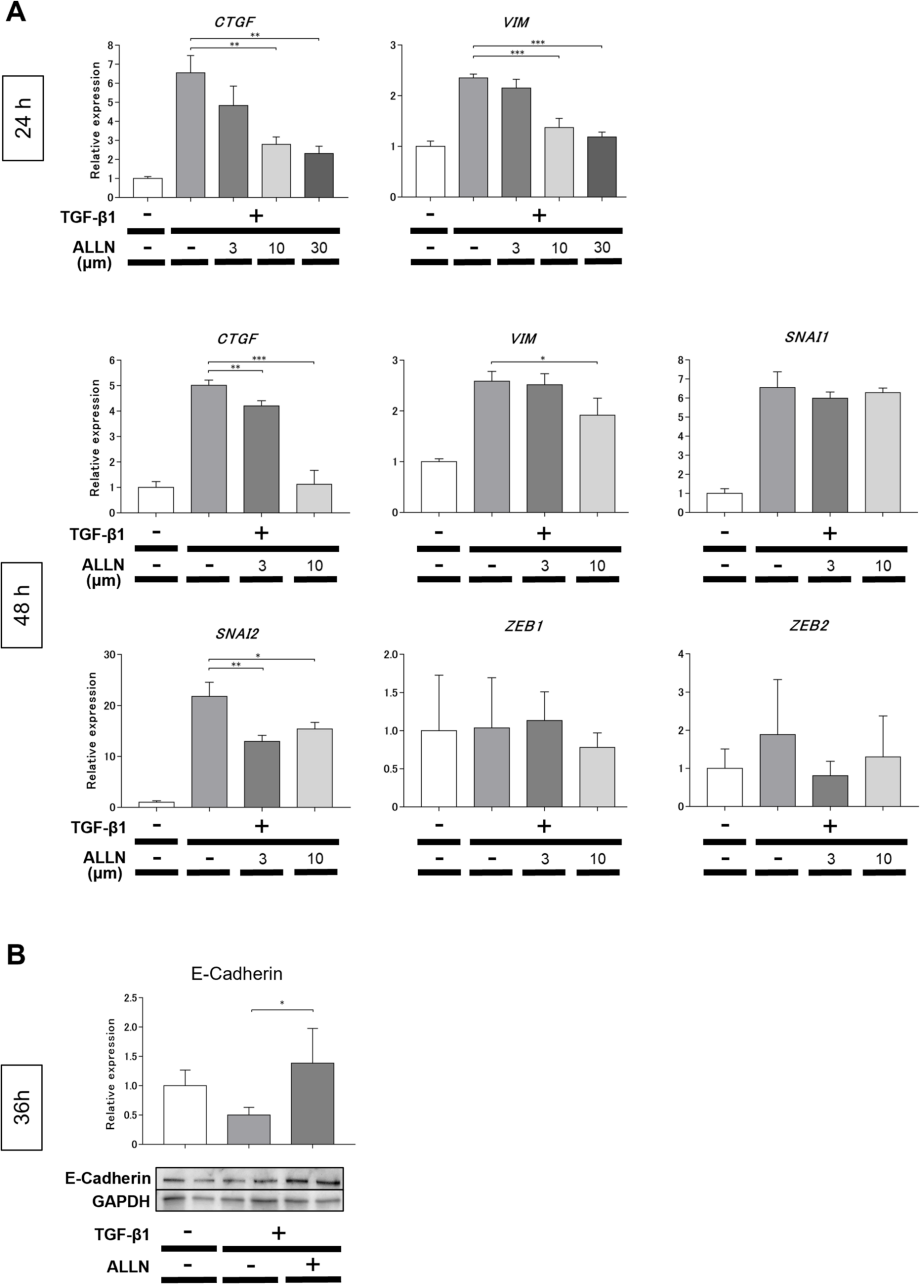
ALLN suppresses EMT of human lung epithelial cells in vitro. Human lung carcinoma epithelial cell line A549 was pretreated with DMSO as control or various concentrations of ALLN diluted in DMSO for 1 h and then stimulated with 10 ng/mL recombinant human TGF-β1. **A** mRNA expression of mesenchymal markers was quantified using real-time RT-PCR at 24 or 48h. Values were normalized to GAPDH levels and are shown as relative expressions; *n* = 3 for each group; * *p* ≤ 0.05; ** *p* ≤ 0.01, *** *p* ≤ 0.001. **B** Protein expression of the epithelial (E-cadherin) marker was quantified by Western blot analyses at 36h. Values were normalized to GAPDH levels and are shown as the relative expression (*n* = 4 for each group). The representative blots are shown. All values represent mean ± SEM; * *p* ≤ 0.05

Other precursors, such as epithelial cells, pericytes, endothelial cells, adipocytes, and bone marrow-derived cells, in addition to resident fibroblasts, may differentiate into myofibroblasts in the fibrotic tissues of SSc, particularly in the lungs [1, 19]. Therefore, we investigated the anti-EMT effects of ALLN on human lung epithelial A549 cells. EMT was induced by incubating A549 cells with TGF-β1 for 24–48 h. ALLN treatment significantly inhibited the mRNA expression of some TGF-β1-induced representative mesenchymal markers, such as connective tissue growth factor (*CTGF*), vimentin (*VIM*), and *SNAI2* induced by TGF-β1 treatment (Fig. 4A). In contrast, the protein expression of E-cadherin, which is a representative epithelial marker, increased following ALLN treatment in TGF-β1-induced A549 cells (Fig. 4B). Therefore, TGF-β1-induced EMT markers were moderated by ALLN treatment in lung epithelial A549 cells.

### Intraperitoneal ALLN attenuated dermal thickening and collagen content in the bleomycin-injected mouse skin

**Fig. 5:**
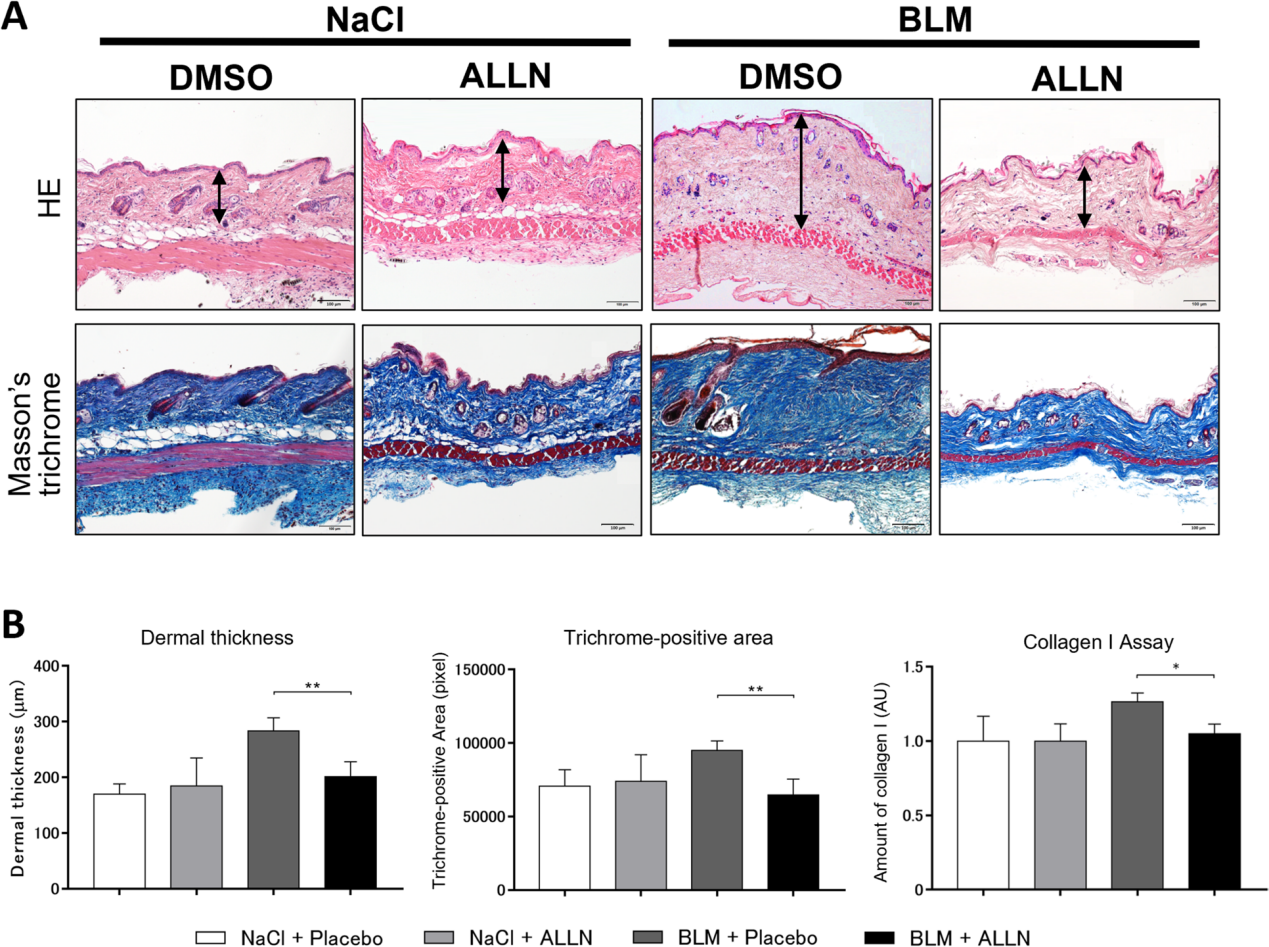
Intraperitoneal ALLN injection ameliorates bleomycin-induced skin fibrosis in mice. **A** The antifibrotic effects of ALLN were analyzed in the back skin of mice receiving daily subcutaneous injections of bleomycin concurrent with a peritoneal injection of ALLN or PBS (3 times per week) for 4 weeks. Representative images of H&E stained (*upper columns*) and Masson’s trichrome stained tissue (*lower columns*) are shown. *Double-headed arrows* indicate the measured length of dermal thickness. Scale bar, 100 μm. **B** Skin fibrosis in bleomycin-injected mice with or without ALLN was compared by determining dermal thickness and ratio of the Masson’s trichrome-stained area; *n* = 5 for each group. Total collagen content in the skin paraffin sections was evaluated by the Sirius Red/Fast Green Collagen Assay; *n* = 3–4 for each group. Values represent mean ± SEM; *, *p* ≤ 0.05

**Fig. 6:**
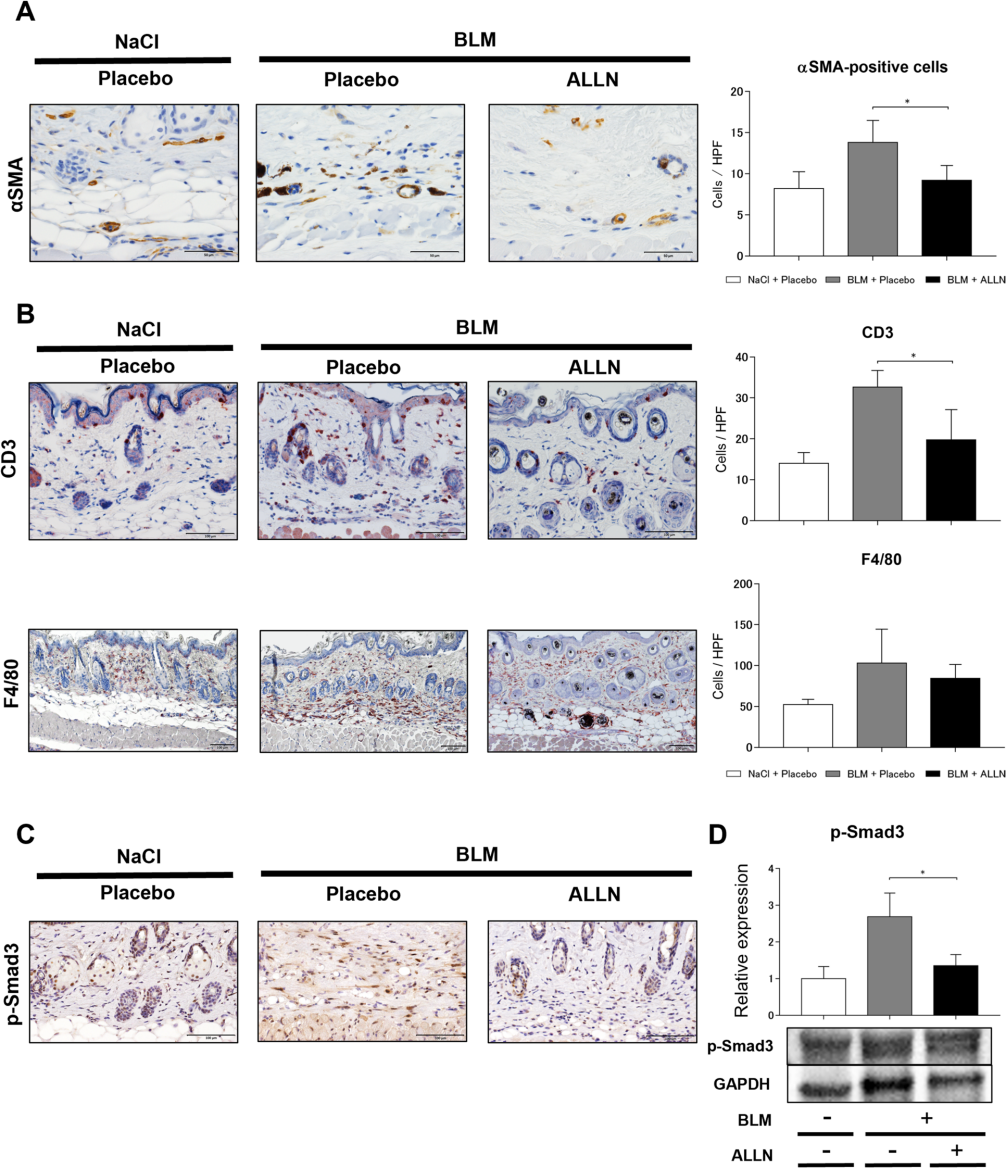
ALLN administration reduced αSMA-positive cells and CD3-positive leukocytes in the skin of daily bleomycin injections. **A** αSMA-positive cells suggestive myofibroblasts were quantified by immunohistochemistry in the skin of mice after 28 days of daily bleomycin injections. Representative images are shown (*left*). Scale bar, 50 μm. Quantitative analysis is shown in the bar graph (*right*). Values represent mean ± SEM; *, *p* < 0.05. **B** The infiltration of CD3-positive T cells and F4/80-positive macrophages were investigated using immunohistochemistry in the skin of mice after 7 days of daily bleomycin injections. Representative images are shown (*left*). Scale bar, 100 μm. Quantitative analysis is shown in the bar graph (*right*). Values represent mean ± SEM; *, *p* ≤ 0.05. **C** The expression of p-Smad cells was evaluated by immunohistochemistry in the skin of mice after 7 days of daily bleomycin injections. Representative immunohistochemistry images are shown in each group; *n* = 5 for each group. Scale bar, 100 μm. **D** Protein expression levels of p-Smad3 were quantified using Western blotting. Values were normalized to GAPDH levels and were shown as relative expressions; *n* = 3 for each group. Values represent mean ± SEM; *, *p* < 0.05

Subcutaneous and intraperitoneal injections of bleomycin and ALLN, respectively, were co-administrated for 4 weeks in C57BL/6J mice. No apparent side effects, including changes in body weight or activity, were observed in any of the mice (data not shown). Histological examination of the bleomycin-injected skin showed a marked increase in dermal thickness, which was significantly reduced by ALLN treatment (Fig. 5A, *upper columns*; Fig. 5B, *left*). In addition, Masson’s trichrome-stained area was significantly reduced in the bleomycin-treated skin from the ALLN-treated group compared with the control group (Fig. 5A, *lower columns* and Fig. 5B, *middle*). These histological changes were consistent with the total collagen content in the skin measured with the Sirius Red/Fast Green Collagen Assay (Fig. 5B, *right*). Furthermore, consistent with these results, αSMA-positive spindle- or star-shaped cells (rather than vessel walls) that appeared to be myofibroblasts were increased in bleomycin-injected skin, which was inhibited by ALLN administration (Fig. 6A).

### ALLN treatment suppressed T cell infiltration in bleomycin-injected mouse skin

The infiltrating cell profile was examined through immunohistochemistry using mouse skin 7 days after bleomycin administration since inflammation preceded tissue fibrosis in our mouse model (Fig. 6B). CD3-positive T cells and F4/80-positive macrophages increased in the skin after bleomycin injection. ALLN administration had little effect on the infiltration of F4/80-positive macrophages compared to the control group, but significantly reduced the infiltration of CD3-positive T cells.

### Intraperitoneal ALLN inhibited p-Smad3 expression in bleomycin-injected mouse skin

We also examined p-Smad3 expression in bleomycin-injected skin for 28 days since ALLN inhibited the protein expression and nuclear transport of p-Smad3 in cultured dermal fibroblasts. Immunohistochemical staining showed that p-Smad3-positive spindle-shaped cells were prominent in bleomycin-injected skin but were suppressed in number by ALLN treatment (Fig. 6C). Quantitative evaluation using Western blotting also showed that the increased amount of p-Smad3 protein in bleomycin-injected skin was significantly decreased by ALLN administration (Fig. 6D). These findings, which were consistent with the in vitro findings, indicated that ALLN disrupts bleomycin-induced TGF-β1 signaling in vivo.

### ALLN treatment reduced the expression of calpain 2 in the bleomycin-injected mouse skin

**Fig. 7:**
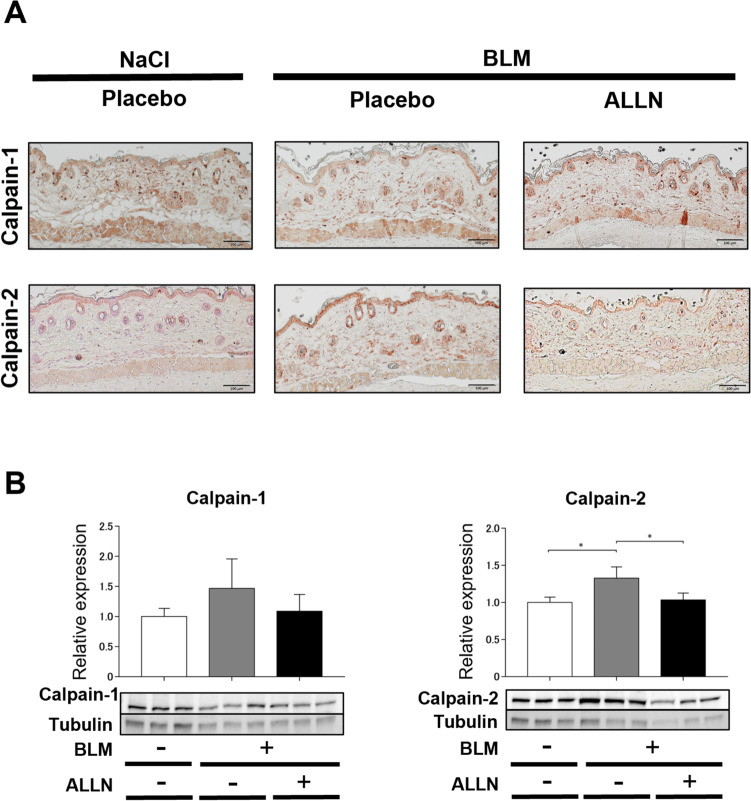
The effect of ALLN treatment on the expression of calpain 1 and 2 associated with EMT in bleomycin-injected mouse skin. **A** Skin sections of placebo-and ALLN-treated mice at day 7 after bleomycin injection were immunostained for calpain 1 and 2. Scale bar, 100 μm. Representative immunohistochemistry images are shown in each group. **B** Protein expression of calpain 1 and calpain 2 in skin samples of placebo- and ALLN-treated mice at day 7 after bleomycin injection were quantitatively analyzed by Western blotting. Values were normalized to tubulin levels and were shown as relative expression values representing mean ± SEM; *n* = 3 for each group; *, *p* < 0.05

Immunohistochemistry showed that calpain 1 and 2 were ubiquitously expressed in various cells of mouse skin (Fig. 7A). Bleomycin injection or ALLN treatment did not affect calpain 1 expression. In contrast, calpain 2 expression increased with bleomycin administration and was inhibited by ALLN treatment. These findings are consistent with the Western blotting results (Fig. 7B). The protein expression of calpain 2, rather than calpain 1, was significantly reduced in the ALLN-treated skin extracts compared with placebo controls.

### Intraperitoneal ALLN improved lung fibrosis induced by subcutaneous bleomycin administration

**Fig. 8:**
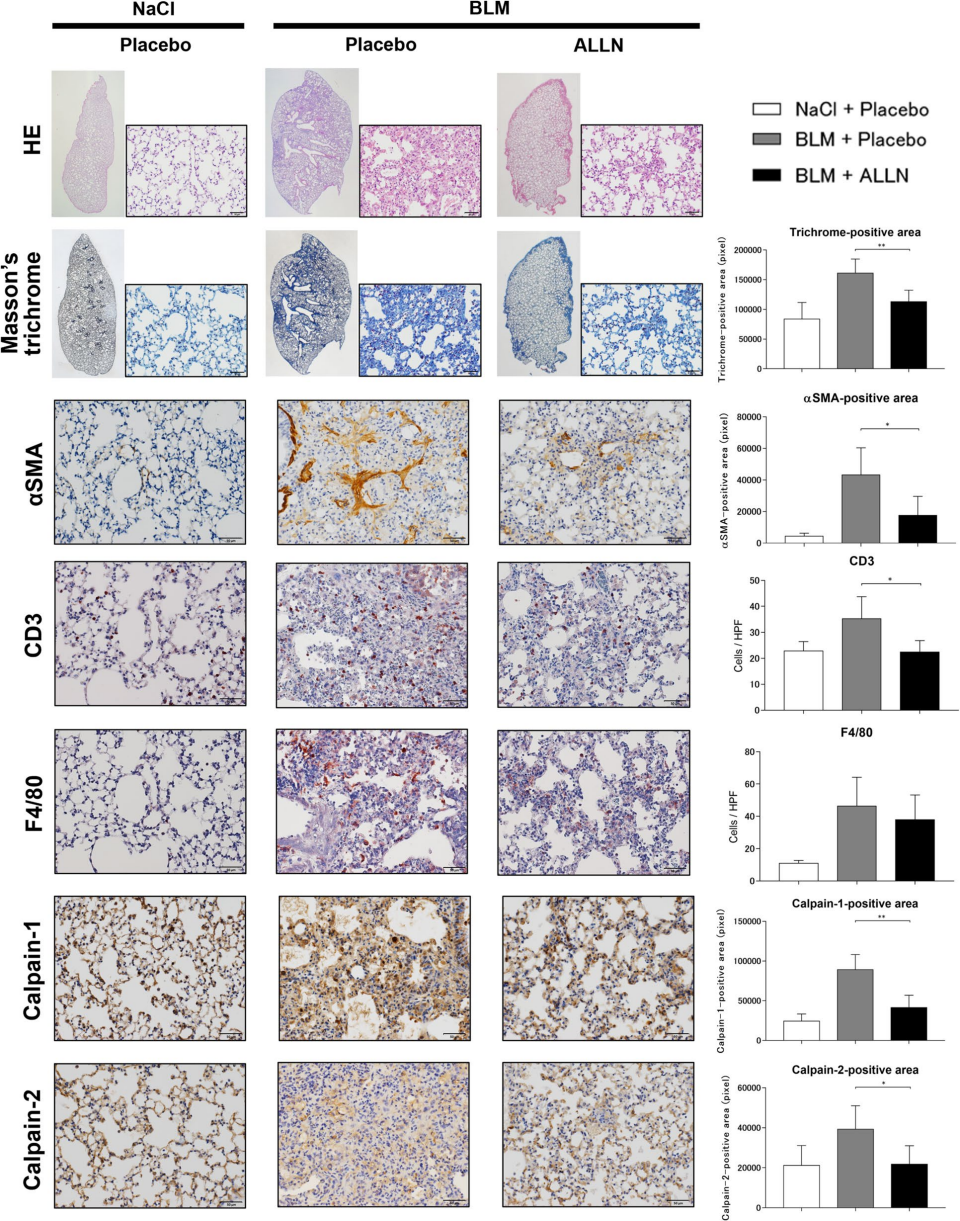
The effect of ALLN treatment on lung fibrosis in subcutaneously bleomycin-injected mouse. Subcutaneous bleomycin injection induces lung inflammation and fibrosis. The antifibrotic effects of ALLN were analyzed in the lung tissues of mice receiving daily subcutaneous injections of bleomycin concurrent with peritoneal injections of ALLN or PBS (three times per week) for 4 weeks. Representative images of H&E and Masson’s trichrome (whole left lung and magnified), αSMA, CD3, F4/80, calpain 1, and calpain 2 staining (magnified) are shown. Scale bar, 50 μm; *n* = 5 for each group

The proportion of stroma with inflammatory cell infiltration increased with a reduction in the functional alveolar structure of the lung parenchyma in the lungs of subcutaneously bleomycin-injected mice compared with that of the PBS-injected mice (Fig. 8). However, intraperitoneal ALLN injection significantly reduced the bleomycin-induced fibrotic stroma of the lungs, which was evaluated using H&E and Masson’s trichrome staining. In addition, the αSMA-positive area, CD3-positive cells, and F4/80-positive cells increased in the lungs after bleomycin injection. However, ALLN treatment significantly reduced the αSMA-positive area and CD3-positive cells. Furthermore, bleomycin administration increased the area positive for calpain 1 or 2, which was also significantly suppressed by ALLN treatment.

## Discussion

In this study, ALLN, one of the cysteine proteinase inhibitors that mainly disrupt calpain activity significantly suppressed p-Smad3 expression, myofibroblast differentiation, and ECM expression in cultured normal human dermal and lung fibroblasts that were induced by TGF-β1. Furthermore, our findings suggest that ALLN inhibits the EMT in TGF-β1-treated human lung epithelial cells. Consistent with these in vitro findings, peritoneal injection of ALLN significantly attenuated the CD3^+^ T cell infiltration and subsequent fibrosis in the skin and lungs of mice subcutaneously injected with bleomycin daily. Bleomycin injection increased the expression of calpain 2 and p-Smad3 in the skin and calpain 1/2 in the lungs; however, these effects were significantly suppressed by ALLN administration. Therefore, ALLN may be useful in inhibiting the progression of skin and lung fibrosis by inhibiting TGF-β1/Smad signaling and other factors in patients with SSc.

ALLN significantly inhibited myofibroblast differentiation and TGF-β1-induced expression and secretion of collagen I and mRNA expression of FN1, ACTA2, SNAIL2, and ZEB1/2 in normal human dermal fibroblasts. Among these, ZEB2 mRNA expression was not increased with TGF-β1 addition and was downregulated by ALLN with or without TGF-β1, suggesting that ALLN suppresses signals other than the TGF-β1-dependent pathway. In addition, p-Smad3 expression and the nuclear transition of mesenchymal transcription markers, such as SNAIL2 and ZEB1, were significantly attenuated by ALLN in normal dermal fibroblasts. ALLN markedly inhibited collagen I production with or without the addition of TGF-β1. This result was consistent with the intracellular protein measurement of collagen I by Western blot. At least according to the Western blot results, ALLN does not affect αSMA or FN1 production in the absence of TGF-β1. Therefore, it seems unlikely that the results of these findings for collagen I are due to drug cytotoxicity. Since ALLN strongly suppresses collagen 1 production by TGF-β1, ALLN may suppress collagen 1 production by TGF-β1 in low concentrations of FBS or by TGF-β1 produced by fibroblasts themselves, even in the group without TGF-β1. However, the possibility that it suppresses collagen I production via cytokines other than TGF-β1 cannot be ruled out.

Consistent with these in vitro data, intraperitoneally injecting ALLN significantly suppressed the development of skin fibrosis, which was induced by daily subcutaneous bleomycin injection. To the best of our knowledge, this is the first report to demonstrate the efficacy of a cysteine proteinase inhibitor in skin fibrosis in an animal model of SSc. In transgenic mice, overexpression of calpastatin, which is an endogenous calpain inhibitor, delayed skin wound healing because of reduced granulation and scar formation [20]. In addition, transgenic overexpression of calpastatin in endothelial cells delays mouse skin wound healing by suppressing PDGF receptor-β signaling in fibroblasts, endothelial cell-driven myofibroblast differentiation, and subsequent fibrogenesis [21]. Moreover, endothelial deletion of calpain 1 and 2 impedes skin wound healing by reducing inflammation and angiogenesis [22]. Therefore, our findings indicate the critical role of calpain in tissue fibrosis and skin remodeling in addition to those of previous reports.

We found that ALLN inhibited the development of subcutaneously injected bleomycin-induced myofibroblasts and subsequent fibrosis in the lungs. Notably, various proteases other than calpains, including serine proteases, cathepsins, matrix metalloproteinases, and members of a disintegrin and metalloproteinase with thrombospondin motifs family, can activate TGF-β1 in vitro and may be implicated in TGF-β1 activation in fibrotic lesions in vivo [23]. Our findings align with a previous report that the cysteine protease inhibitor calpeptin, which mainly inhibits calpain activity, was effective in a mouse model of lung fibrosis induced by intraperitoneal administration of bleomycin [13]. Lung fibrosis in these models was induced via bleomycin in the circulating blood. In another study, intratracheal administration of bleomycin, the most common route of bleomycin-induced lung fibrosis, elevated the expression levels of calpain 1 and 2, which was subsequently suppressed by calpeptin [24]. Calpeptin reduced collagen expression and impaired the TGF-β1-induced differentiation of normal human lung fibroblasts into αSMA-expressing myofibroblasts similar to our results for dermal and lung fibroblasts [20]. These findings suggest that calpains play a crucial role in lung fibrosis development.

Subcutaneously injecting bleomycin causes inflammation that precedes fibrosis in the skin and lungs [15, 16]. However, administering ALLN significantly reduced CD3-positive T cells rather than F4/80-positive macrophages in the affected skin and lungs. Although the exact role of calpain in chemokine-induced chemotaxis for many cell types is not yet known, one may suggest the involvement of cell type-dependent signaling [25]. Regarding T cell recruitment, the inhibitory effect of calpeptin, a cysteine proteinase inhibitor, has been observed in chemokine CCL2-mediated chemotaxis of Jurkat T cells [26]. However, it is not clear why ALLN suppresses the infiltration of only T cells and not macrophages in bleomycin-induced skin and lung fibrosis mouse models.

Unfortunately, no excellent method of measuring calpain activity has been established. Therefore, it can only be estimated that changes in calpain expression reflect changes in activity. It has been reported that ALLN inhibits not only calpain activation but also its expression in rat sinusoidal endothelial cells [27]. In a mouse model of bleomycin-induced pulmonary fibrosis, calpeptin downregulated calpain 1/2 mRNA expression in the lungs [13]. In the current study, subcutaneous injection of bleomycin promoted calpain 2 and 1/2 expressions in the skin and the lungs, respectively, whereas ALLN treatment suppressed it. In contrast, injection of bleomycin or ALLN administration did not significantly affect calpain 1 expression in the skin. Moreover, bleomycin increased the expression of p-Smad3 in the skin, whereas ALLN treatment significantly reduced this effect. Li et al. [14] demonstrated that bleomycin induces calpain and TGF-β1 activation which triggers TGF-β1 Smad and non-Smad (Akt) signaling pathways via TGF-β receptor in primary human lung fibroblasts. TGF-β1 also activates calpain causing a mutual interaction and subsequently enhancing canonical TGF-β1 signaling that results in excessive collagen production [14]. These previous in vitro findings and our results demonstrate an inhibitory effect of ALLN on TGF-β1 signaling in bleomycin-induced fibrotic tissues.

Recent studies using single-cell RNA sequences indicate that myofibroblasts are mostly derived from resident fibroblasts in the skin of SSc [19, 28]. However, other precursor cell sources, including epithelial cells, pericytes, endothelial cells, adipocytes, and bone marrow-derived cells, may contribute to fibrosis by differentiating into myofibroblasts [1]. Although EMT may be involved in lung fibrosis, the conversion of epidermal keratinocytes into mesenchymal cells has not been considered one of the main mechanisms of skin fibrosis in SSc [19]. Increased expression of calpain 1, combined with the reduced expression of E-cadherin in the lungs of patients with idiopathic pulmonary fibrosis, indicates a relationship between ubiquitous calpains and EMT in pulmonary fibrosis [24]. A previous study demonstrated that ubiquitous calpain inhibition using calpeptin suppresses EMT and fibrotic changes in the lungs both in vivo and in vitro [24]. Another manuscript indicates that calpain 1 could regulate EMT in TGF-β1-treated A549 lung epithelial cells via the PI3K/Akt signaling pathway [29]. In this study, we evaluated the effects of ALLN on the TGF-β1-induced EMT phenotype in A549 cells. ALLN restored the expression of the attenuated epithelial marker, E-cadherin, and weakened that of the mesenchymal markers, such as CTGF, VIM, and SNAIL2. Therefore, at least in vitro, ALLN may be as protective as calpeptin in preventing EMT in lung epithelial cells*.*

This study had some limitations. First, ALLN inhibits cysteine cathepsins such as cathepsin B and cathepsin L in addition to calpain 1 and 2. Therefore, it is unclear whether the inhibition of skin and lung fibrosis by ALLN is solely due to calpain inhibition. The previous literatures [30, 31] indicate that upregulated cathepsin B and cathepsin L in endothelial cells can induce vascular injury, but downregulated these cathepsins in the dermal fibroblasts or skin have fibrotic effects. The mRNA expression of cathepsin B and L is decreased in the skin of bleomycin-induced mouse model. Catepshin L is also downregulated in lung tissue and lung fibroblasts in SSc and is thought to have an inhibitory effect on fibrosis [32]. Therefore, it is unlikely that the anti-fibrotic effect of ALLN in the present study is due to the inhibition of cathepsin B and/or L. Second, regarding the effect of ALLN on TGF-β1 signaling, we investigated only canonical Smad2/3 signaling rather than non-canonical signaling pathways such as PI3K/Akt signaling [33]. Third, additional investigations using other SSc mouse models are required to confirm their clinical applications. Inhibition of calpain has been reported to inhibit the proliferation of vascular smooth muscle cells in diabetes [34]. In addition, studies using mouse models of hypoxia-induced pulmonary hypertension and pulmonary arterial smooth muscle cells suggest that calpain-1 may be important for hypoxia-inducible factor-1α-mediated vascular remodeling and fibrosis [35]. Therefore, although we were not able to evaluate vascular injury in the present study, further studies are needed to determine the utility of ALLN for vascular injury associated with SSc.

## Conclusions

In conclusion, the therapeutic effect of ALLN on skin and lung fibrosis was remarkable in a subcutaneous bleomycin injection-induced SSc mouse model. Therefore, drugs that inhibit in vivo calpain activity, such as ALLN, may be valuable candidates for treating fibrotic disorders, such as SSc.

## Data Availability

The datasets used and/or analyzed during this study are available and can be obtained from the corresponding author upon reasonable request.

## Abbreviations

αSMA: α-Smooth muscle actin
ALLN: N-Acetyl-Leu-Leu-norleucinal
BLM: Bleomycin
COL1A2: Collagen type I alpha 2
CTGF: Connective tissue growth factor
DAPI: 4,6-Diamidino-2-phenylindole
DMSO: Dimethyl sulfoxide
EMT: Epithelial-mesenchymal transition
FN1: Fibronectin 1
GAPDH: Glyceraldehyde-3-phosphate dehydrogenase
H&E: Hematoxylin and eosin
PBS: Phosphate-buffered saline
RT-PCR: Reverse transcription-polymerase chain reaction
SEM: Standard error of the mean
SNAIL2: Snail family transcriptional repressor 2
TGF-β1: Transforming growth factor beta 1
VIM: Vimentin
ZEB1: Zinc-finger-enhancer binding protein 1
